# Does size affect the prognosis of resectable thymoma beyond the eighth edition TNM?

**DOI:** 10.1111/1759-7714.14255

**Published:** 2021-12-20

**Authors:** Yen‐Chiang Tseng, Han‐Shui Hsu, Yi‐Hsuan Lin, Yen‐Han Tseng, Chih‐Wen Shu, Yih‐Gang Goan, Ching‐Jiunn Tseng

**Affiliations:** ^1^ Division of Thoracic Surgery, Department of Surgery Kaohsiung Veterans General Hospital Kaohsiung Taiwan; ^2^ Institute of Clinical Medicine, National Yang‐Ming University Taipei Taiwan; ^3^ Division of Thoracic Surgery, Department of Surgery Taipei Veterans General Hospital Taipei Taiwan; ^4^ School of Medicine, National Yang‐Ming University Taipei Taiwan; ^5^ Institute of Emergency and Critical Care Medicine, National Yang‐Ming University Taipei Taiwan; ^6^ Department of Family Medicine, School of Medicine National Yang‐Ming University Taipei Taiwan; ^7^ Department of Public Health, College of Public Health National Taiwan University Taipei Taiwan; ^8^ Department of Chest Medicine Taipei Medical University – Shuang Ho Hospital Taipei Taiwan; ^9^ Institute of Biopharmaceutical Sciences, National Sun Yat‐Sen University Kaohsiung Taiwan; ^10^ Department of Biomedical Science and Environmental Biology Kaohsiung Medical University Kaohsiung Taiwan; ^11^ Department of Medical Education and Research Kaohsiung Veterans General Hospital Kaohsiung Taiwan

**Keywords:** thymoma, TNM staging system, tumor size

## Abstract

**Background:**

Thymoma is a type of rare mediastinal tumor whose clinical characteristics and indicators of prognosis are poorly understood. This single‐institution retrospective study aimed to assess the predictive value of tumor, node, metastasis (TNM) staging incorporating tumor size in predicting the risk of thymoma recurrence after resection.

**Methods:**

Four binary logistic regression models were developed. Models I and II included median tumor size and TNM stage, respectively. Model III included the above two variables. Model IV was model III containing these two variables and their interaction terms. All models were adjusted for WHO histological type, operational time, and adjuvant therapy.

**Results:**

A total of 276 patients with a median age of 51.0, including 21 patients with thymoma recurrence, were included in this study. Models II or III showed a lower ‐2LogL and higher AUC (0.735 and 0.738 vs. 0.576) with significantly better discrimination than model I, and model III and model II shared similar discrimination. In model III, TNM stage was positively correlated with thymoma recurrence. The recurrence risk of patients with TNM stage IV was significantly higher than those with TNM stage I (OR of 11.03, *p* = 0.022). No significant correlation between the tumor size and recurrence risk *(p* = 0.779) and no interaction was found between medium tumor size and TNM stage in model IV.

**Conclusions:**

This study suggests that the prediction contribution of the TNM stage combined with tumor size is similar to the TNM stage alone for tumor recurrence in patients with thymoma after surgical resection.

## Graphical abstract

A multiple logistic regression model was used to measure the associations of tumor, node, metastasis (TNM) stage and tumor size with recurrence of thymoma and thymic carcinoma. Models I and II included medium tumor size and TNM stage, respectively. Model III included the two variables. Model IV was model III containing interaction terms (TNM stage x medium tumor size). The tesults show that medium tumor size, TNM stage III, or TNM stage IV were significantly associated with thymoma recurrence risk, respectively. The additive effect is absent between tumor size and TNM staging.

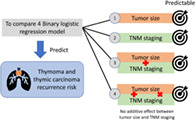

## INTRODUCTION

Thymoma is a type of rare mediastinal tumor with specific clinical and morphological characteristics.^1^ The incidence of thymic epithelial tumors is approximately 0.15 cases per year in the United States.^2^ This type of cancer is very heterogeneous with a broad range of morphological appearances and immunological abnormalities. Due to the rarity of these tumors, the clinical characteristics and indicators of prognosis are not well understood.^1^ Clinically, thymomas generally result in pleural dissemination.^3^ The treatments for thymoma include surgery, radiation, and chemotherapy.^4^, ^5^, ^6^ However, an optimal therapeutic strategy still remains controversial.^7^

More than 10 different stage classification systems have been proposed and utilized for thymoma.^8^, ^9^, ^10^, ^11^, ^12^, ^13^ Previous studies have indicated that the best predictors of outcomes for thymoma are the World Health Organization (WHO) histological classification system, the modified Masaoka staging system, and the tumor, node, and metastasis (TNM)‐staging system.^14^, ^15^, ^16^, ^17^, ^18^, ^19^, ^20^, ^21^, ^22^, ^23^ Furthermore, in recent years, the modified Masaoka staging and TNM‐staging systems have both been reported to be more reliable, useful, and comparable for staging and treatment.^17^, ^24^ The TNM‐staging system has been reported to be applicable for stage‐adapted therapy and prognosis prediction for overall and recurrence‐free survival and is significantly correlated with the WHO histological classification system.^25^, ^26^ It also shows more clinically relevant differentiation than the modified Masaoka staging system.^27^

The TNM‐staging system is composed of three factors (tumor, node, metastasis) which defines the overall stage of the tumor and its classification is similar to cancers staged from I–IV.^28^ Tumor size has been included in the definition of TNM staging for solid tumors, and smaller tumor size and complete resection has been reported to be associated with better survival in thymoma patients, showing its potential to be an independent prognostic factor.^29^ However, no study to date has revealed the relationship of tumor size and TNM staging and the prediction value when combining them for thymoma prognosis. This study evaluated the hypothesis that combining tumor size and TNM‐staging, both considered independent prognostic factors of survival, might increase the ability to predict tumor relapse or disease‐free survival following tumor resection in patients with thymic epithelial tumors and more available for the clinician in diagnosis.

## METHODS

This was a single‐site retrospective chart review at Taipei Veterans General Hospital that utilized data from December 1997 to March 2013. The study was performed in accordance with the declaration of Helsinki. The Institutional Review Board of the Taipei Veterans General Hospital approved the protocol and granted an exemption from informed consent (201 208 010 BC).

### Patient study and design

Patients who underwent surgery for thymoma with complete clinical follow‐up and histopathological data were enrolled. Patients who had neoadjuvant therapy, no biopsy data, WHO histological type C (before 2004) thymic carcinoma (after 2004), and patients without surgical margin were excluded. Two experienced pathologists (H‐L K and T‐Y C) reviewed all the slides of thymoma or thymic carcinoma.

The primary endpoints were tumor recurrence and the predictive value of tumor recurrence by tumor size and TNM staging. The TNM system is composed of three factors defining the overall stage of the tumor and its classification is similar to cancers staged from I–IV, with stage IV being the most severe.^28^ Stage 0 is carcinoma in situ, which is not considered as cancerous but might potentially subsequently become cancer. TNM classification is: (1) stage I ‐ localized cancer, without lymph node involvement; (2) stage II ‐ locally advanced cancer, without lymph node invasion; (3) stage III ‐ locally advanced cancer, with lymph node involvement; and (4) stage IV ‐ metastatic cancer, with distal metastasis.^28^

#### Multivariable regression models

We applied a backward deletion approach (*p* < 0.05) to identify all covariates as potential confounders in the multivariable model. Then, a multiple logistic regression model with adjusting confounders was implemented to measure the associations of TNM stage and tumor size with recurrence of thymoma. Models I and II included medium tumor size and TNM stage, respectively. Model III included the two variables. Model IV was model III containing interaction terms (TNM stage x medium tumor size). Odds ratio (OR) and 95%confidence interval (CI) were estimated to quantify the strength of association. The predictive performances of TNM stage and/or tumor size are expressed as discrimination (area under the receiver operating characteristic curve, AUC) and calibration. Calibration represents how recurrence predictions resemble the observed recurrence, which was measured by the Hosmer and Lemeshow goodness‐of‐fit test.

### Statistical analysis

Initially, the Shapiro‐Wilk test was used to check the normal distribution of continuous data, such as age, operational duration, and median tumor size. Continuous data with normal distribution were presented as mean ± SD and performed with a Student's *t*‐test. Otherwise, continuous data without normal distribution were presented as median (25th–75th percentile；Q1–Q3) and performed by Wilcoxon rank sum test. In addition, categorical variables were computed frequency and percentage and performed by chi‐squared test or Fisher's exact test. All statistics were two‐sided and performed using SAS statistical software (version 9.4).

## RESULTS

**FIGURE 1:**
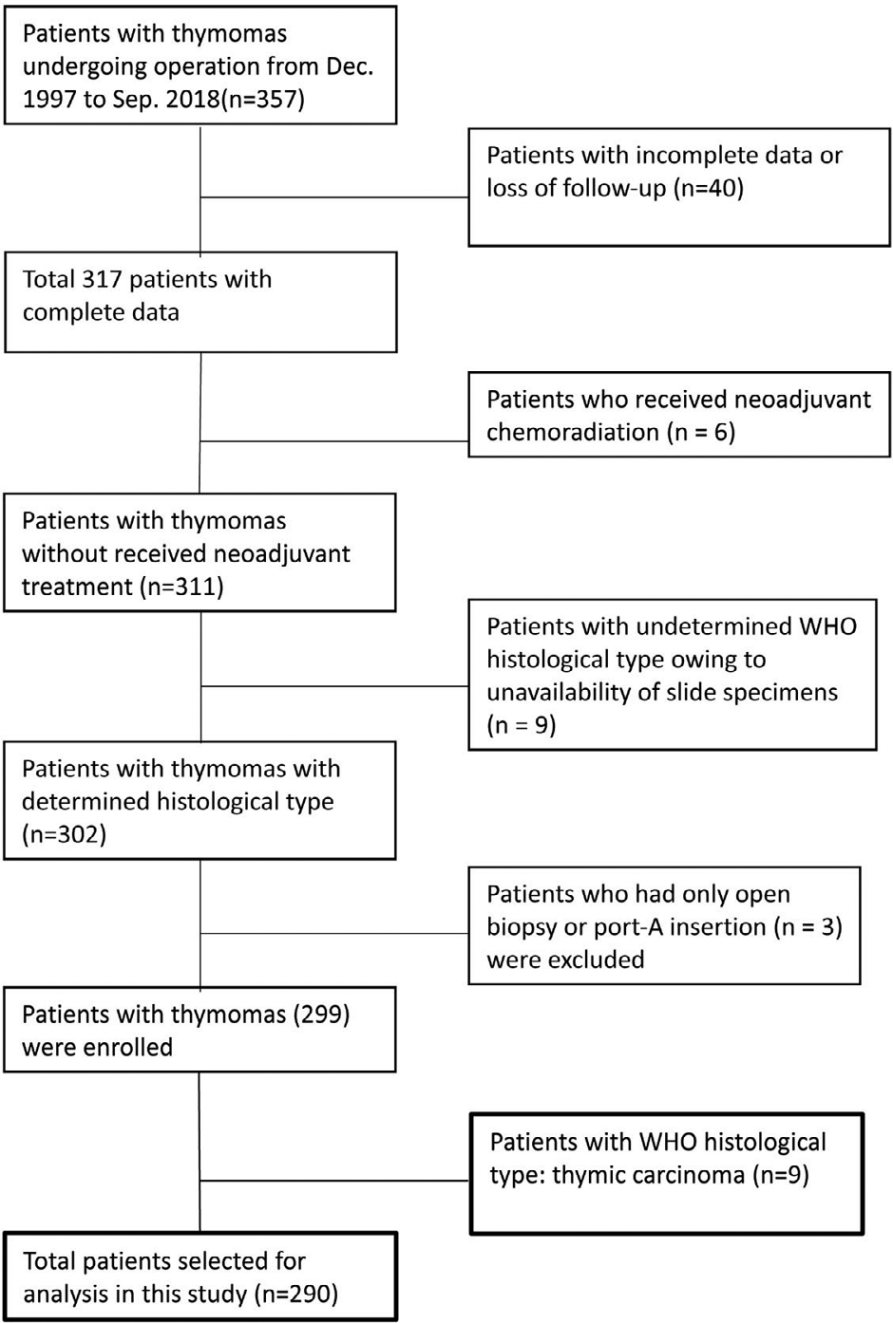
Flow diagram of patient data

A total of 357 patients were initially enrolled in this study. After excluding 40 patients with incomplete data, six patients who received neoadjuvant therapy, 12 patients without pathological data, nine patients who were WHO histological type ‘C’, and 14 patients without surgical margins were excluded (Figure 1). A total of 276 patients were finally included and analyzed.

The characteristics of 276 patients are shown in Table 1. The median age, medium tumor size, and operational time of this study population were 51.0 (IQR = 19.5, Q1–Q3: 43.5–63.0) years, 5.7 (IQR = 3.0, Q1‐Q3: 4.5–7.5) cm, and 180.0 (IQR = 117.5, Q1–Q3: 102.5–220.0) minutes. More than half of the patients were female (55.07%) with tumors of WHO histological type AB, B1, or B2 (74.63%). Around 76.09% of patients had TNM stage I tumors. Median sternotomy (55.07%) and video‐assisted thoracic surgery (33.33%) were the most commonly performed surgery, and 95.17% were margin‐free. More than half of patients did not undergo adjuvant therapy (60.14%). The length of stay in intensive care unit was 1–2 days in 50% patients and 3–7 days in 17.39% patients. Postoperative myasthenia gravis and recurrence were reported in 27.54% and 7.61% of patients, respectively. Table 1 shows that some variables are related to recurrence, including WHO histological type, TNM stage, operational time, and adjuvant therapy. The recurrence rate of patients who suffered from WHO histological type B3 was 22.45% and higher than the others (*p* < 0.001). Furthermore, the TNM stage IV patients who underwent adjuvant therapy were more likely to experience a recurrence (recurrence rate: 62.50% and 14.55%). The operational time of patients with recurrence is longer than patients without recurrence (*p* < 0.001). According to the backward deletion approach, the covariates were identified confounders in the final model, including WHO histological type, operational time, and adjuvant therapy.

**TABLE 1:** Patient characteristics

Variables	Total (*N* = 276)	Recurrence		*p*‐value
		Yes (*N* = 21)	No (*N* = 255)	
Age, years	51.0 (43.5–63.0)	54.0 (43–63)	51 (45–64)	0.255^a^
Gender				0.513^b^
Male	124 (44.93%)	8 (6.45%)	116 (93.55%)
Female	152 (55.07%)	13 (8.55%)	139 (91.45%)
Medium tumor size, cm	5.7 (4.5–7.5)	6.0 (5.0–8.0)	5.4 (4.3–7.5)	0.250^a^
Medium tumor size (categories)				0.274^c^
<8	214 (77.54%)	14 (6.54%)	200 (93.46%)
≧8	62 (22.46%)	7 (11.29%)	55 (88.71%)
WHO histological type				**0.001** ^**c**^
A	21 (7.61%)	1 (4.76%)	20 (95.24%)
AB	92 (33.33%)	2 (2.17%)	90 (97.83%)
B1	56 (20.29%)	4 (7.14%)	52 (92.86%)
B2	58 (21.01%)	3 (5.17%)	55 (94.83%)
B3	49 (17.75%)	11 (22.45%)	38 (77.55%)
TNM stage				**<0.001** ^**c**^
I	210 (76.09%)	8 (3.81%)	202 (96.19%)
II	37 (13.41%)	3 (8.11%)	34 (91.89%)
III	21 (7.61%)	5 (5%)	16 (76.19%)
IV	8 (2.90%)	5 (62.50%)	3 (37.50%)
***Operational characteristics***				
Operational time, minutes	180.0 (102.5–220.0)	270.0 (180.0–360.0)	175.0 (100.0–215.0)	**<0.001** ^a^
Surgical approach				0.128^b^
Median sternotomy	152 (55.07%)	14 (9.21%)	138 (90.79%)
Thoracotomy	32 (11.59%)	4 (12.50%)	28 (87.50%)
VATS	92 (33.33%)	3 (3.26%)	89 (96.74%)
Length of ICU stay (day)				
0	87 (31.52%)	4 (4.60%)	83 (95.40%)	0.472^c^
1–2	138 (50.00%)	14 (10.14%)	124 (89.86%)
3–7	48 (17.39%)	3 (6.25%)	45 (93.75%)
>7	3 (1.09%)	0 (0.00%)	3 (100.00%)
***Treatment features***				
Adjuvant therapy				**<0.001** ^**b**^
None	166 (60.14%)	5 (3.01%)	161 (96.99%)
Yes	110 (39.86%)	16 (14.55%)	94 (85.45%)
***Postoperative outcomes***			
Myasthenia gravis				0.912^**b**^
No	200 (72.46%)	15 (7.50%)	185 (92.50%)
Yes	76 (27.54%)	6 (7.89%)	70 (92.11%)

**Abbreviations:** ICU, intensive care unit; VATS, video‐assisted thoracic surgery.

*Note*: Continuous data are presented as median (IQR; interquartile: 25th‐75th percentile).

^a^ Using Wilcoxon rank sum test.

^b^ Using Chi‐square test.

^c^ Using Fisher's exact test.

**FIGURE 2:**
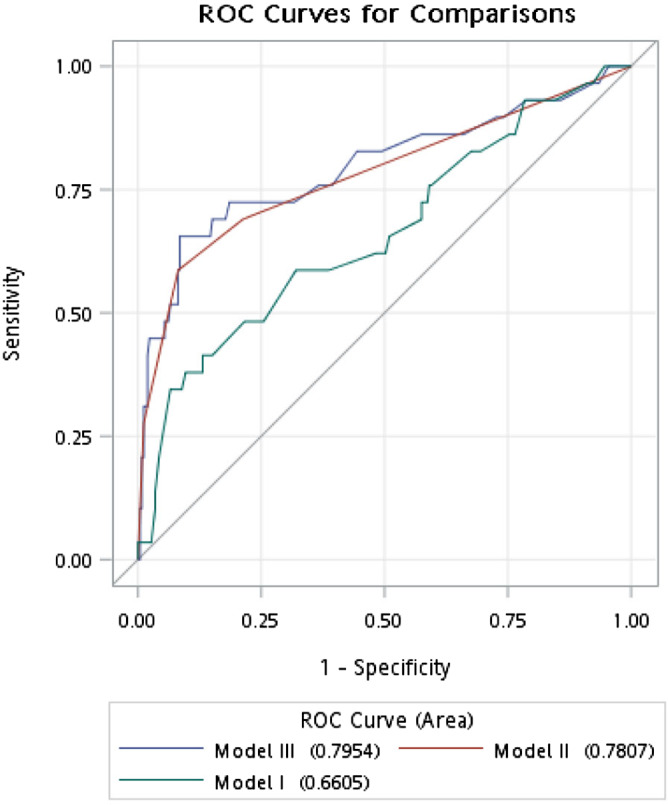
The predicted recurrence probability on model I – III

The results of four models presenting recurrence risk are summarized in Table 2. The AUC and difference of models I to III are summarized in Table 3. Among the four models, models II and III were better than model I due to the smaller ‐2LogL and higher AUCs (0.735 and 0.738 vs. 0.576). Model III had significant better discrimination than model I (AUC difference = 0.160, *p* = 0.015). Models III and II had similar discrimination (AUC difference = −0.003, *p* = 0.921) (Table 3 and Figure 2).

**TABLE 2:** Results of four recurrence models

	Model I		Model II		Model III		Model IV	
	aOR (95% CI)	*p‐*value	aOR (95% CI)	*p‐*value	aOR (95% CI)	*p‐*value	aOR (95% CI)	*p‐*value
**Medium tumor size (cm)**	1.00 (0.83, 1.19)	0.964	‐		0.92 (0.75, 1.12)	0.404	0.96 (0.71, 1.30)	0.779
**TNM stage**								
I			Ref		Ref		Ref	
II	‐		2.48 (0.55, 11.27)	0.240	2.84 (0.60, 13.39)	0.186	0.09 (0.00, 12.22)	0.333
III	‐		1.99 (0.41, 9.71)	0.396	2.23 (0.45, 11.11)	0.328	4.76 (0.15, 149.37)	0.375
IV	‐		7.57 (1.22, 47.15)	**0.030**	11.03 (1.39, 69.47)	**0.022**	322.98 (1.03, >999)	**0.049**
**Medium tumor size × TNM stage**								
I	‐		‐		‐		Ref	
II	‐		‐		‐		1.54 (0.86, 2.73)	0.1442
III	‐		‐		‐		0.91 (0.57, 1.44)	0.6811
IV	‐		‐		‐		0.66 (0.33, 1.31)	0.2304
‐2Log L	110.41		104.89		104.18		100.91	
**Hosmer‐Lemeshow test**	‐	0.502	‐	0.964	‐	0.828	‐	0.676

**Abbreviations:**−2LogL: (−2) × Log‐likelihood ratio; aOR, adjusted odds ratio; CI, confidence interval.

*Note*: Adjusted OR were adjusted for WHO histological type B, operational time, and adjuvant therapy. Bold value denotes statistically significant, *p* < 0.05.

**TABLE 3:** The area under the receiver operating characteristic curve (AUC) and difference in models I to III

Model	AUC (95% CI)	*p*‐value
III	0.735 (0.597, 0.873)	
II	0.738 (0.618, 0.859)	
I	0.576 (0.441, 0.710)	
III–I	0.160 (0.031, 0.289)	**0.015**
II–I	0.163 (0.010, 0.315)	**0.037**
III–II	−0.003 (−0.064, 0.058)	0.921

*Note*: Bold value denotes statistically significant, *p* < 0.05.

In Model III, the TNM stage was positively correlated to recurrence of thymoma. The risk of recurrence of the patients with TNM stage IV was significantly higher than that of the patients with TNM stage I (stage IV: OR = 11.03, 95% CI: 1.39–69.47, *p* = 0.022). There was no significant correlation between tumor size and recurrence risk (*p* = 0.779) and no interaction was found between medium tumor size and TNM stage in model IV. Thus, the prediction contribution of the TNM stage combined with tumor size is similar to the TNM stage alone for tumor recurrence in patients with thymoma after surgical resection.

## DISCUSSION

This study assessed the predictive value of TNM staging incorporated tumor size in thymoma recurrence after resection. Four models were developed using binary logistic regression for evaluating the relationship of tumor size and TNM staging in predicting recurrence. The results identified that WHO histological type B3, TNM stage IV, and adjuvant therapy were risk factors associated with thymoma recurrence. Similar prediction trends of thymoma recurrence were found when using the TNM staging before or after incorporating the tumor size without any interaction between these two parameters. The results revealed the absence of additive effect between tumor size and TNM staging in predicting the thymoma recurrence. Therefore, we suggest that more attention and follow‐up strategies are necessary for the thymoma patients with initial WHO histological type B3, TNM stage IV, and adjuvant therapy, even after radical resection surgery.

According to previous studies, the inclusion of tumor size in primary staging systems for prognosis prediction of resectable thymoma is still controversial. In the IASLC/ITMIG thymic epithelial tumors staging project, proposals for the T component for the forthcoming (eighth) edition of the TNM staging of malignant tumors state that tumor size is not included. Nicholson et al. did not find survival differences according to the tumor size in patients with completely resected thymic tumors based on the ITMIG database. The retrospective analysis identified that 10 cm was the only valid cut point among the cohort received tumor resection and the best cut point was 9.5 cm.^30^ Overall, survival curves demonstrated a difference in the resected cohort due to a difference in outcomes among incompletely resected patients; however, there was no difference among R0 patients. This retrospective study analyzed 5796 cases and it is the largest cohort known today.^30^ A well‐known retrospective study by Ruffini et al. included more than 2000 patients from the European Society of Thoracic Surgeons' (ESTS) database; it identified that tumor size was neither a predictor of overall survival nor of recurrent‐free survival.^30^ Using tumor size as a continuous variable (1‐cm increase), the authors found that a larger tumor size is a predictor of incomplete resection and increases the risk of recurrence.^30^

Some authors identified tumor size as an independent predisposing factor. A small cohort by Fukui et al.^31^ of Nagoya University Graduate School of Medicine in Japan recently reported that recurrence‐free survival was significantly worse in patients with tumors >4.0 cm in diameter than those with smaller tumors. Moreover, a multivariate analysis showed that tumor size >4.0 cm was an independent prognostic factor (HR 5.236 [1.170–23.256]; *p* = 0.03) for recurrence‐free survival in patients with thymic carcinoma (*n* = 21) or thymic neuroendocrine tumor (*n* = 9) but not in patients with thymoma (*n* = 124).^31^

Wright et al. found that recurrence rate correlated with tumor size and that there was an increased incidence of recurrence at 8 cm (<8 cm, 1.8%; ≥8 cm, 28%).^32^ In addition, the study by Harnath et al. found that thymic epithelial tumors <8.5 cm had an independent favorable prognosis.^33^ Two previous studies have indicated poorer prognosis with a cutoff of >6 cm.^14^, ^34^ Roden and his colleagues^35^ reported that patients surgically treated for thymic epithelial neoplasm at Mayo Clinic from 1942 to 2008 were staged according to the modified Masaoka staging system and were included for retrospective analysis. Masaoka stage predicted outcomes independent of all histopathological classifications and resection status and strongly correlated with the proposed Moran stage (correlation coefficient, 0.95). Thymoma size was a prognostic parameter for OS independent of any histopathological classification but not superior to the modified Masaoka staging.^35^ Safieddine et al.^36^ presented a retrospective analysis from a single institute that included 262 patients. The analysis of multivariate models included completeness of resection and excluded Masaoka stage. It showed that tumor size was a significantly poor predisposing factor. Among the 262 surgically resected cases, adverse prognostic factors included incomplete resection, larger sized tumors (> 7 cm), and higher Masaoka stage.

Several reasons may have caused the failure to observe a significant improvement in predicting relapse in thymoma patients when combining TNM staging and tumor size as prognostic parameters for tumor recurrence. First, this study did not take the step‐up effect of tumor size on tumor recurrence into account. Second, the models in this study were not adjusted for different follow‐up durations, adjunct chemo‐ or radiotherapies, and WHO histological type. Third, this was a retrospective, single‐center study with inherent limitations. Finally, the total number of patients is relatively small for validation of the proposed TNM system.

In conclusion, TNM staging and tumor size have previously been independent prognostic factors for recurrence and survival in thymoma. However, this study found no improvement in the prognosis prediction value of the TNM staging system incorporated tumor size in thymoma.

## CONFLICT OF INTEREST

The authors confirm that there are no conflicts of interest
